# Systemic inflammation suppresses spinal respiratory motor plasticity via mechanisms that require serine/threonine protein phosphatase activity

**DOI:** 10.1186/s12974-021-02074-6

**Published:** 2021-01-19

**Authors:** Arash Tadjalli, Yasin B. Seven, Raphael R. Perim, Gordon S. Mitchell

**Affiliations:** Breathing Research and Therapeutics Center, Department of Physical Therapy and The McKnight Brain Institute, College of Public Health & Health Professions, University of Florida, 1225 Center Drive, PO Box 100154, Gainesville, FL 32610 USA

**Keywords:** Inflammation, Lipopolysaccharide, Motor neuron, Protein phosphatases, Plasticity, Intermittent hypoxia, Spinal cord, Breathing, Phosphorylation

## Abstract

**Background:**

Inflammation undermines multiple forms of neuroplasticity. Although inflammation and its influence on plasticity in multiple neural systems has been extensively studied, its effects on plasticity of neural networks controlling vital life functions, such as breathing, are less understood. In this study, we investigated the signaling mechanisms whereby lipopolysaccharide (LPS)-induced systemic inflammation impairs plasticity within the phrenic motor system—a major spinal respiratory motor pool that drives contractions of the diaphragm muscle. Here, we tested the hypotheses that lipopolysaccharide-induced systemic inflammation (1) blocks phrenic motor plasticity by a mechanism that requires cervical spinal okadaic acid-sensitive serine/threonine protein phosphatase (PP) 1/2A activity and (2) prevents phosphorylation/activation of extracellular signal-regulated kinase 1/2 mitogen activated protein kinase (ERK1/2 MAPK)—a key enzyme necessary for the expression of phrenic motor plasticity.

**Methods:**

To study phrenic motor plasticity, we utilized a well-characterized model for spinal respiratory plasticity called phrenic long-term facilitation (pLTF). pLTF is characterized by a long-lasting, progressive enhancement of inspiratory phrenic nerve motor drive following exposures to moderate acute intermittent hypoxia (mAIH). In anesthetized, vagotomized and mechanically ventilated adult Sprague Dawley rats, we examined the effect of inhibiting cervical spinal serine/threonine PP 1/2A activity on pLTF expression in sham-vehicle and LPS-treated rats. Using immunofluorescence optical density analysis, we compared mAIH-induced phosphorylation/activation of ERK 1/2 MAPK with and without LPS-induced inflammation in identified phrenic motor neurons.

**Results:**

We confirmed that mAIH-induced pLTF is abolished 24 h following low-dose systemic LPS (100 μg/kg, i.p.). Cervical spinal delivery of the PP 1/2A inhibitor, okadaic acid, restored pLTF in LPS-treated rats. LPS also prevented mAIH-induced enhancement in phrenic motor neuron ERK1/2 MAPK phosphorylation. Thus, a likely target for the relevant okadaic acid-sensitive protein phosphatases is ERK1/2 MAPK or its upstream activators.

**Conclusions:**

This study increases our understanding of fundamental mechanisms whereby inflammation disrupts neuroplasticity in a critical population of motor neurons necessary for breathing, and highlights key roles for serine/threonine protein phosphatases and ERK1/2 MAPK kinase in the plasticity of mammalian spinal respiratory motor circuits.

## Introduction

Neuro-inflammation is an important feature in the pathogenesis and progression of numerous clinical disorders [1–7]. Neuro-inflammation can modulate various biological processes that are essential for normal neural function such as synaptic plasticity [8, 9]. For example, inflammatory processes can elicit maladaptive forms of neural plasticity such as chronic pain [10–12], or undermine beneficial plasticity such as learning and memory or motor plasticity [13–16]. Despite the known impact of neuro-inflammation on certain forms of plasticity, its impact on plasticity of neural networks giving rise to essential behaviors such as breathing is poorly understood [17, 18]. Adaptive plasticity within respiratory motor circuits is an important contributor to the preservation and/or restoration of respiratory function in face of disease and/or injury [19–22]. Since virtually all clinical disorders that compromise breathing are associated with inflammation, inflammatory processes may impair robust and plastic motor responses that are critical for maintenance of adequate breathing and blood gas regulation.

Low-dose systemic lipopolysaccharide (LPS) delivery—pathogen-associated molecular patterns that signal through innate immune toll like receptor 4 (TLR4)—transiently increases inflammatory gene expression in ventral cervical spinal cord segments associated with the phrenic motor nucleus—a major spinal respiratory motor pool that drives contractions of the diaphragm muscle [23]. LPS also blocks the expression of a form of phrenic motor plasticity referred to as phrenic long-term facilitation (pLTF) [17, 23]. pLTF is a commonly studied form of spinal respiratory motor plasticity that is characterized by a long-lasting increase in ventilation or phrenic nerve motor activity induced by exposures to acute intermittent, but not continuous hypoxia [24–28]. LPS effects on pLTF expression are reversed by nonsteroidal anti-inflammatory drugs (ketoprofen), conforming the role of inflammation in the mechanisms undermining pLTF [23]. However, our current understanding of the cellular signaling mechanisms by which inflammatory insults impair respiratory phrenic motor plasticity remains limited. Therefore, our goal here was to reduce this knowledge gap by advancing our mechanistic understanding concerning how LPS-induced inflammation undermines moderate acute intermittent hypoxia-induced (mAIH) pLTF and, for the first time, determine whether systemic LPS effects the expression of plasticity in other respiratory motor pools, such as brainstem hypoglossal motor neurons that innervate important respiratory muscles of the upper airways.

Phrenic LTF requires cervical spinal Gq protein-coupled serotonin type 2A and 2B receptor activation [29], NADPH oxidase activity/reactive oxygen species formation [30], ERK1/2 MAPK activity [31], PKC-θ activity [32], new BDNF protein synthesis, and TrkB signaling within phrenic motor neurons [28, 33]. Further, pLTF is constrained by okadaic acid-sensitive serine/threonine protein phosphatases (PPs) during continuous, but not intermittent hypoxia [34]. In our model, serine/threonine PPs are important regulators of moderate AIH-induced pLTF. Regulation of protein phosphorylation is determined by the balance of kinase and phosphatase activities [35, 36]. Serine/threonine PPs regulate multiple signaling cascades that are essential for synaptic plasticity, and are in turn, activated by inflammation [37]. For example, these PPs exert significant regulatory control over ERK 1/2 MAPK activity—key enzyme necessary for the expression of AIH-induced pLTF expression [31, 38–40].

Protein phosphatases often serve as an important crosstalk link between MAPK pathways that often reciprocally oppose one another [41–43]. For example, upregulation of p38 MAPK signaling (a key molecule that initiates and responds to inflammation) following inflammatory stimuli [44] increases serine/threonine PP activity and indirectly dephosphorylates specific residues in the ERK1/2 MAPK activation loop [45–48]. In the phrenic motor system, pro-inflammatory stimuli such as chronic intermittent hypoxia (modeling sleep apnea) that lead to deleterious effects activate p38 MAPK in phrenic motor neurons and suppresses AIH-induced pLTF by a p38 MAPK-dependent mechanism [49]. We reasoned that serine/threonine PP activation may represent an essential “next step” in this important regulatory process. We propose to test the specific hypothesis that low-dose systemic LPS blocks mAIH-induced pLTF expression by a mechanism that requires cervical spinal okadaic acid-sensitive serine/threonine PP activity. We further hypothesize that LPS-induced systemic inflammation blocks mAIH-induced ERK1/2 MAPK phosphorylation/activation within phrenic motor neurons, thereby undermining pLTF. Further, we predict that LPS delivery activates p38 MAPK within phrenic motor neurons, providing supporting evidence that ERK1/2 MAPK vs p38 MAPK balance is a key regulator of phrenic motor plasticity. Lastly, we test the hypothesis that LPS prevents the expression of plasticity in other respiratory motor pools, such as the hypoglossal motor nucleus that innervate upper airway respiratory muscles.

## Methods

Experiments were conducted on adult male Sprague Dawley rats (Envigo, Colony 208A), weighing 373 ± 3 g (78 rats in total; weights ranged from 331 to 439 g, 373 (± standard error); ~ 3.5–4 months of age). All procedures were approved by the Animal Care and Use Committee at the University of Florida (Protocol # 201408657). Rats had access to food and water ad libitum and were kept in a 12-h daily light-dark cycle.

### Drugs and vehicles

LPS (*E. Coli* 0111:B4, catalog number L3024) and Okadaic acid (Okadaic Acid Sodium Salt, Catalog number 459620) were obtained from Sigma Aldrich (St. Louis, MO). On arrival, LPS was dissolved in sterile phosphate-buffered saline (PBS) and aliquots of the stock solution were frozen at – 20 °C. Okadaic acid was dissolved in artificial CSF (aCSF; 120 mm NaCl, 3 mm KCl, 2 mm CaCl, 2 mm MgCl, 23 mm NaHCO3,10 mm glucose bubbled with 95% O2–5% CO_2_), and aliquots were also frozen at − 20 °C. On the day of the experiments, drugs were diluted (LPS in sterile saline and Okadaic acid in freshly made aCSF) to achieve the desired final concentration/dose. LPS (100 μg/kg) or vehicle (sterile saline) was administered via intraperitoneal injections (lower left abdominal quadrant) 24 h prior to beginning an experiment. We chose a low LPS dose since our goal was to characterize the impact of low-grade inflammation on respiratory motor plasticity. This dose is relevant since low grade systemic inflammation is characteristic of many clinical disorders that compromise breathing.

Okadaic acid was administered intrathecally at the level of the cervical spinal cord (cervical segment 2; see the “Surgical procedures” section below) at a concentration of 25 nM approximately 30 min before experimental protocols commenced. Okadaic acid is a potent inhibitor of serine/threonine protein phosphatase 1 and 2A and displays more than 100,000,000-fold selectivity over other protein phosphatases such as PP2B and PP2C [50, 51]. The concentration (25 nM) was based on literature and its well-documented pharmacology. At the same concentration, we have shown that serine/threonine PP are active in the spinal region containing phrenic motor neurons since they constrain pLTF during continuous (but not intermittent) hypoxia [34]. Immunohistochemistry and activity assays to confirm expression and activity of these phosphatases in ventral spinal segments were associated with the phrenic motor nucleus [34].

### Surgical procedures

Rats were first anesthetized with 3% inspired isoflurane in an induction chamber and then through a nose cone (60% O2 balance N2) while surgery was performed. After confirming absence of the foot-pinch withdrawal reflex, a midline ventral cervical incision was made in the neck; the trachea was exposed and sectioned below the larynx; a tracheal tube (polyethylene catheter; PE 240; Intramedic, MD, USA) was inserted into the trachea to deliver isoflurane and controlled gases via artificial ventilation (2.5% isoflurane mixed in 60% O2/ balance N2). Artificial ventilation was achieved by using a rodent ventilator (tidal volume = 0.7 ml/g; Rodent Respirator model 683, Harvard Apparatus, South Natick, MA). A rapidly responding flow-through CO2 analyzer (Capnogard, Novametrix, Wallingford, CT) was placed on the expired tubing of a Y-tube connected to the tracheal cannula for monitoring end-tidal PCO2 (PetCO2). The tail vein was then cannulated (24 gauge, Surflo, Elkton, MD, USA) so that rats could be slowly converted from isoflurane to urethane anesthesia (2.1 mg/kg; i.v.). The conversion from isoflurane to urethane was carried out at least an hour before the start of experimental protocols. The foot-pinch withdrawal reflex response was used to test the adequacy of anesthesia; supplemental anesthetic was given as required. Once urethane conversion was complete, fluids were given through the same tail vein cannula to maintain acid-base balance (1.5–2.5 ml/h, started approximately 1 h after the beginning of surgery; 1:4 solution of 8.4% sodium bicarbonate mixed in standard lactated Ringer’s solution). Body temperature was monitored with a rectal thermometer (Fischer Scientific, Pittsburgh, PA, USA) and maintained (37.5 ± 1 °C) with a custom-made heated surgical table.

Rats were bilaterally vagotomized at the mid-cervical region to prevent entrainment of respiratory motor output with the ventilator. Rats were paralyzed with pancronium bromide (2 mg/kg; Sigma-Aldrich, St. Louis, MO) to prevent desynchrony between the ventilator and spontaneous respiratory movements. A flexible polyethylene catheter (PE 50; Intramedic MD, USA) was inserted into the femoral artery. The distal end of the arterial catheter was connected to a pressure transducer (Grass Instruments) to monitoring arterial blood pressure and withdrawal. The same arterial line was also used for withdrawing blood samples (70 μl samples) for blood gases measurements (i.e., PaO2 and PaCO_2_) and acid-base balance using a blood gas analyzer (ABL 90 Flex, Radiometer, Copenhagen, Denmark).

Using a dorsal approach, the left hypoglossal and phrenic nerves were isolated, cut distally, and de-sheathed. Nerves were kept moist by a saline-soaked cotton ball until ready to be placed in custom-made suction recording electrodes (see the “Electrophysiological recordings and measurements” section below) to record respiratory neural activity. Long-term facilitation (LTF) is observed as an increase in inspiratory motor activity of phrenic (innervating the diaphragm) and hypoglossal nerves (innervating the tongue muscle) following AIH. Activities recorded from these nerves serve as indices of spinal cord versus cranial brainstem respiratory motor output, respectively. Therefore, in addition to the phrenic nerve in this study, we also record from the hypoglossal nerve because (1) we wanted to characterize the impact of systemic inflammation on the expression of brainstem respiratory motor plasticity for the same since upper airway motor plasticity is hypothesized to stabilize breathing and preserve airway patency and (2) to take advantage of the anatomical separation between brainstem hypoglossal versus spinal phrenic motor neurons to ensure that intrathecal spinal drug injections (see below) do not spread beyond the spinal cord at effective concentrations.

To deliver drugs to the cervical spinal cord, a laminectomy was performed over the C2 vertebrae, a small hole was cut in the dura near the junction of C2 and C3 spinal segments, and a flexible silicone catheter (0.6 mm outer diameter; Access Technologies) was fed through the hole, advancing the catheter tip to the rostral end of C3. The catheter was connected to a 50-μl Hamilton microinjector syringe containing either aCSF (vehicle) or dugs dissolved in aCSF.

### Electrophysiological recordings and measurements

Phrenic and hypoglossal nerves were placed in custom-made glass suction electrodes filled with 0.9% saline. Nerve activity was amplified (10 K, A-M systems, Everett, WA), filtered (bandpass 100-5000 Hz), integrated (time constant, 50 milliseconds), digitized (Micro1401, Cambridge Electronic Design, UK), and analyzed using Spike 2 software (Cambridge Electronic Design, UK; version 8.08). Following surgical preparations and conversion to urethane anesthesia, rats were allowed a minimum of 1 h to stabilize before beginning an experimental protocol. Inspiratory phrenic and hypoglossal activities served as indices of respiratory motor output. Measurements of inspiratory phrenic and hypoglossal burst frequency and amplitude were assessed in 1-min bins immediately prior to each blood sample during baseline, during hypoxic episodes, and at 30, 60, and 90 min post AIH. Measurements were also made at equivalent time points in time-matched control experiments without AIH.

### Immunohistochemical experiments

#### In vivo retrograde labeling of phrenic motor neurons

Since the aim of our immunohistochemical experiments was to quantify phosphorylation levels of ERK1/2 MAPK and p38 MAPK in or near identified phrenic motor neurons (see the “Study 5—Does LPS affect mAIH-induced phrenic motor neuron ERK1/2 MAPK phosphorylation?” section below), we retrogradely labeled phrenic motor neurons. To do this, rats (29 rats in total) received Cholera toxin β subunit (CtB) administration via intrapleural injections (Mantilla et al., 2009) made at least 10 days prior to experiments. Rats were anesthetized with isoflurane (1.5–2% in 100% O_2_) via a nose cone, and the left and the right rib cage areas were shaved to expose the skin. After disinfecting the area with alcohol and chlorhexidine wipes (Covidien llc, MA), 12.5 μL of CtB (0.2% w/v CtB dissolved in sterile H_2_O; Calbiochem, MA) was injected into the right and left thoracic cavities at the 5th intercostal space using a Hamilton syringe (6 mm deep using 22s gauge semi-blunt needle tip; 12.5 μL on each side). Following injections, anesthesia was discontinued, and rats were monitored for any signs of respiratory complications; no complications were observed in any of the rats. This method allows phrenic nerve axon endings to retrogradely transport CtB into phrenic motor neurons. An antibody against CtB can then be used for immunofluorescent labeling of phrenic motor neurons within the spinal cord (see below).

#### Immunofluorescence

Conventional double immunofluorescence labeling was employed to stain for CtB and phospho-p38 MAPK. All rats underwent the same surgical procedure and were exposed to the same anesthetic protocols (e.g., isoflurane to urethane convention) as previously described (see the “Surgical procedures” section). This was done to mimic the same conditions compared to rat groups exposed to mAIH or normoxia during neurophysiological recordings, but not used for immunostaining. Urethane-anesthetized rats were perfused transcardially with ice-cold phosphate-buffered saline (PBS, pH 7.4) followed by 4% buffered paraformaldehyde (PFA, pH 7.4). Cervical segment of the spinal cord containing phrenic motor neurons (C3-C5) was excised, post-fixed in 4% paraformaldehyde overnight, and cryoprotected in 30% sucrose at 4 °C. Forty-micrometer transverse sections were cut using a freezing microtome (Leica SM 2010R, Germany) and stored in anti-freeze solution (− 20 °C) until the day of staining. Transverse tissue sections were numbered sequentially and 2 sections per spinal segment (C3, C4 and C5) were used to represent C3-C5 for each animal (total of 6 spinal sections per animal). Free-floating sections were washed in 0.1 M PBS containing 0.1% Triton-X100 (PBS-TX; 3 × 5 min washes). Tissues were then blocked with 5% normal donkey serum for 1 h at room temperature to block non-specific binding sites. Staining was performed by incubating free-floating tissues with primary antibodies against CtB (goat host, 1:2500, EMD Millipore) and phospho-p38 MAPK (rabbit host, 1:500, Cell Signaling Technology, Inc. Product ID# 4511) over night at 4 °C (diluted in 2.5% donkey serum in PBS). Tissues were then washed and then incubated with secondary antibodies (1 h room temperature in PBS-0.1% TX) to label CtB (Donkey anti-goat Alexa Fluor 488, 1:1000, Invitrogen) and phospho-p38 MAPK (Donkey anti-rabbit, 1:500, Alexa Fluor 594, Invitrogen). Sections were then immediately washed and mounted using VectaShield Hardset mounting medium (Vector Laboratories, UK).

#### Immunohistochemistry

To visualize phospho-ERK1/2 MAPK in CtB-labeled phrenic motor neurons, a combination of 3,3’-diaminobenzidine (DAB)-peroxidase and immunofluorescence double labeling technique was utilized. Specifically, DAB-Peroxidase-based staining was first used to label for phospho-ERK1/2 MAPK and then was followed by immunofluorescence to stain for CtB in the very same tissue sections. To stain for phospho-ERK1/2, free-floating tissues were first washed in 0.1 M PBS containing 0.1% Triton-X100 (PBS-TX; 3 × 5-min washes). They were then incubated in PBS containing 1% hydrogen peroxide for 30 min to reduce background endogenous peroxidase activity. Sections were then washed in PBS-TX (3 × 5 min) and then blocked with 5% normal donkey serum at room temperature for 1 h. Tissues were then incubated with anti-phospho-ERK1/2 antibody (rabbit host, 1:500, Cell Signaling Technology, Inc. Product ID# 4370S) over night at 4 °C (diluted in 2.5% donkey serum in PBS). The sections were then washed and incubated with biotinylated secondary donkey anti-rabbit antibody (1:1000, ThermoFisher Scientific, Product ID# A16027) for 1 h at room temperature. They were then washed and conjugated with avidin–biotin complex (VECTASTAIN Elite ABC kit PK-6100, Vector Laboratories, Burlingame, CA) followed by treatment with 3,3′-DAB-peroxidase solution (Vector Laboratories, UK) according to the instruction provided by the manufacturer. Tissues were then immediately washed in PBS and then processed for immunofluorescence staining against CtB as described in the previous section (see the “Immunofluorescence” section above). The final product was a double labeling to visualize phospho-ERK1/2 MAPK in CtB-positive phrenic motor neurons. Controls without either primary or secondary antibodies were run concurrently to ensure specific labeling.

### Experimental protocols

At least 1 h after conversion to urethane anesthesia, apneic and recruitment CO_2_ thresholds of respiratory nerve activity was determined by lowering inspired CO_2_ (or increasing ventilation rate in some cases) levels until rhythmic respiratory nerve activity ceased. After ~ 60 s, inspired CO_2_ was slowly increased until rhythmic respiratory nerve bursts resumed. The end-tidal PCO_2_ at which respiratory nerve activity stopped and then resumed were considered the apneic and recruitment thresholds, respectively. Baseline conditions were then established by holding end-tidal PCO_2_ ∼ 2 mmHg above the recruitment threshold and allowing sufficient time to establish stable nerve activity (> 25 min). During baseline recordings, an arterial blood sample was taken to document baseline blood gas levels. Arterial PCO_2_ was maintained isocapnic (± 1.5 mmHg) with respect to this baseline value throughout experiments by actively manipulating inspired carbon dioxide concentration and/or ventilation rate. Baseline oxygen levels (∼ 60% inspired oxygen, balance N2 and CO_2_) were maintained for the duration of experiments except for hypoxic challenges; target arterial PaO_2_ levels during hypoxic episodes were 35–50 mmHg.

#### Study 1—Does mAIH induce phrenic and hypoglossal LTF in healthy rats?

To confirm that mAIH can induce both phrenic and hypoglossal LTF, sham vehicle-treated rats were exposed to 3, 5-min episodes of isocapnic (± 1.5 mmHg CO_2_ from baseline) hypoxia (∼ 11.5% inspired O_2_) separated by 5-min intervals of baseline O_2_ conditions (*n* = 6 rats). After the third hypoxic episode, rats were returned to baseline inspired O_2_ levels and biological variables were further recorded for the next 90-min. Additional groups of time-matched control rats without AIH exposure were used to demonstrate that respiratory activity remained stable throughout the recording period (*n* = 5 rats). These rats were pretreated with spinal intrathecal injections of the vehicle (1 × 12 μl injection; aCSF; delivered over 1 min at C3-C5 spinal segment) as a vehicle control for rats receiving their respective spinal injections of drugs dissolved in aCSF (see the “Study 2—Does cervical spinal okadaic-acid delivery affect LTF in normal sham control rats?” section below).

#### Study 2—Does cervical spinal okadaic-acid delivery affect LTF in normal sham control rats?

The main goal of the study was to determine if cervical spinal inhibition of serine/threonine protein phosphatase 1/2A (via okadaic acid) could restore phrenic motor plasticity following LPS-induced systemic inflammation. Before doing so, we ensured that okadaic acid itself did not interfere mAIH-induced respiratory LTF in normal sham control rats. Thus, a group of sham vehicle-treated rats was given cervical spinal intrathecal okadaic acid (1 × 12 μl injection of okadaic acid dissolved in aCSF) 30 min before mAIH (*n* = 7 rats). An additional rat group was injected spinally with okadaic acid, but without mAIH exposure. This group served as a time control for okadaic acid-treatment in normal healthy rats (*n* = 6 rats).

#### Study 3—Does LPS-induced systemic inflammation block hypoglossal and phrenic LTF?

Before determining whether spinal okadaic acid restores phrenic LTF in LPS-treated animals (see the “Study 4—Does cervical spinal okadaic acid restore phrenic LTF in LPS-treated rats?” section below), we first confirmed LPS blocks mAIH-induced pLTF. In addition, we wanted to determine for the first time if systemic LPS affects the expression of hypoglossal LTF. Rats were injected with systemic LPS, and 24 h later exposed to mAIH as described above (see the “Study 1—Does mAIH induce phrenic and hypoglossal LTF in healthy rats?” section; *n* = 7 rats). An additional group of time-matched LPS-injected rats without mAIH exposures were used to demonstrate that respiratory nerve activity remained stable throughout the neurophysiological recording period (*n* = 6 rats). These rats were pretreated with intrathecal injections of vehicle (1 × 12 μl injection; aCSF; delivered over 1 min at C3-C5 spinal segment) as a control group to compare with rats receiving spinal drugs (see the “Study 5—Does LPS affect mAIH-induced phrenic motor neuron ERK1/2 MAPK phosphorylation?” section below).

#### Study 4—Does cervical spinal okadaic acid restore phrenic LTF in LPS-treated rats?

In study 3, we determined that systemic LPS blocks the expression of both phrenic and hypoglossal mAIH-induced LTF (see the “Results” section). Thus, we determined if cervical spinal okadaic acid restores phrenic LTF in LPS-treated rats. We expect hypoglossal LTF to remain absent in the same rats since our pharmacological manipulation was presumably restricted to the cervical spinal cord. LPS pre-treated rats were administered cervical spinal intrathecal okadaic acid (1 × 12 μl injection dissolved in aCSF) 30 min before exposures to mAIH (*n* = 6 rats). An additional group of LPS-pretreated rats was injected with okadaic acid alone without exposures to mAIH (*n* = 6 rats). This group served as a time control experiment for okadaic acid-treatment in LPS-injected rats.

#### Study 5—Does LPS affect mAIH-induced phrenic motor neuron ERK1/2 MAPK phosphorylation?

Although ERK1/2 MAPK is a critical enzyme required for mAIH-induced pLTF, it is unknown if systemic inflammation affects mAIH-induced ERK1/2 MAPK activity (phosphorylation) within phrenic motor neurons. Since mAIH is a physiological stimulus known to induce respiratory motor plasticity (e.g., pLTF), we determined (1) if mAIH increases ERK 1/2 MAPK phosphorylation within phrenic motor neurons of sham vehicle-treated rats and (2) if LPS-induced systemic inflammation prevents increased phrenic motor neuron ERK 1/2 MAPK phosphorylation in response to mAIH. Rats underwent the same surgical and anesthetic procedures described above. Phrenic nerves were isolated, and after establishing baseline nerve activities, rats were exposed to mAIH. Biological variables were recorded for 15 min post-mAIH and rats were then immediately transcardially perfused to enable immunohistochemistry (*n* = 5 rats). We sacrificed rats 15 min after mAIH because of the known dynamics and time course for ERK1/2 MAPK phosphorylation/activation [52]. For example, various stimuli induce a biphasic ERK1/2 MAPK activation, with a rapid, strong burst of kinase activity peaking at 10–15 min, followed by a second wave of lower but sustained activity persisting for hours [53, 54]. Thus, 15 min post-mAIH stimulation, we would expect to see maximum ERK 1/2 MAPK phosphorylation levels. Results were compared to spinal tissues collected at an equivalent time point from control rats that were not exposed to mAIH (*n* = 6 rats). Another rat group was injected with systemic LPS, and 24 h later, identical procedures were performed. Spinal tissue was collected from LPS-pretreated rats 15 min post-mAIH (*n* = 6 rats). Spinal tissue was also collected from separate time-matched LPS-treated rats that were not exposed to mAIH (*n* = 6 rats).

#### Study 6—Does LPS increase p38 MAPK phosphorylation levels in phrenic motor neurons?

Since p38 MAPK has been implicated in the regulation of inflammatory signaling, we evaluated LPS effects on p38 MAPK phosphorylation levels in identified phrenic motor neurons via immunofluorescence. Cervical spinal tissue sections were harvested from sham vehicle-treated rats 24 h following vehicle injections (*n* = 3 rats). Spinal tissues were also collected from a separate rat group receiving systemic LPS injections (*n* = 3 rats). Double immunofluorescence labeling was used to stain for phospho-p38 MAPK in CtB-labeled phrenic motor neurons. Image analysis was performed to quantify differences in phrenic motor neuron phosphorylated p38 MAPK levels in vehicle versus LPS-treated rats.

For studies 5 and 6, images were captured using a microscope designed for both brightfield and fluorescence microscopy (BZ-X710, Keyence Co., Osaka, Japan). All images were captured at × 20 magnification. CtB immunolabeling was detected using a GFP filter (BZ-X, model no: OP-87763) at an excitation filter of 472/30 nm. p38 MAPK labeling was detected using a TexasRed filter (BZ-X, model no: OP-87765) at an excitation filter range of 624/40 nm. Since phospho-ERK1/2 MAPK was stained using a biotinylated secondary antibody designed for (DAB)-peroxidase reaction (non-fluorescence tagged antibody), brightfield microscopy at 20X was used to capture ERK 1/2 MAPK immuno-reactivity (dark brown spots indicating positive immune reaction).

### Data analysis

Respiratory nerve activities were analyzed using Spike 2 software (Cambridge Electronic Design, UK; version 8.08). Integrated phrenic and hypoglossal nerve inspiratory burst amplitudes were averaged over 1-min bins at each experimental time point. Specifically, activities were analyzed during baseline, hypoxia, and at 30, 60 and 90-min post-hypoxia. Changes (Δ) in nerve burst amplitudes were normalized and reported as percentage change from baseline (baseline = 0). Therefore, any value below zero is a decrease whereas values above zero are increases relative to baseline. Burst frequencies (breaths per minute) were also analyzed and presented as a change from baseline in the number of breaths per minute (Table 3). Respiratory activities were analyzed at equivalent time points in time-matched control animals that were not exposed to hypoxia. We also measured and analyzed mean arterial pressure (MAP), arterial CO_2_ pressure (PaCO_2_), arterial O_2_ pressure (PaO_2_), and standard base excess (SBEc) at the indicated time points (Tables 1, 2, and 3). Values for these variables were not normalized and were presented as absolute values. Statistical comparisons between treatment groups for all the variables were made via two-factor ANOVA with a repeated measures design. Individual comparisons were made using the Fisher LSD post hoc test (SigmaPlot version 14; Systat Software Inc., San Jose, CA, USA). Differences between groups were considered significant if *P* < 0.05. All values are expressed as means ± S.E.M.

**Table 1:** Physiological variables at baseline, hypoxia, and the post-hypoxic period in rats receiving intrathecal cervical spinal injections of the vehicle, or okadaic acid

Experimental groups	PaCO_**2**_ (mmHg)	PaO_2_ (mmHg)	SBEc mmol/L
**Sham + vehicle + mAIH**			
Baseline	44.0 ± 1.5	315 ± 11	1.13 ± 0.2
Hypoxia	43.6 ± 1.4	40 ± 1.0*	1.35 ± 0.4
30 min	43.9 ± 1.7	256 ± 16*	1.20 ± 0.4
60 min	44.2 ± 1.3	279 ± 2*	1.75 ± 0.5
90 min	44.5 ± 1.5	265 ± 9*	1.55 ± 0.5
**LPS + vehicle + mAIH**			
Baseline	42.4 ± 1.2	318 ± 10	0.3 ± 0.6
Hypoxia	42.3 ± 1.0	42 ± 1.3*	0.8 ± 0.5
30 min	42.4 ± 1.1	270 ± 18*	2.1 ± 0.7
60 min	42.2 ± 1.3	276 ± 11*	1.6 ± 0.9
90 min	42.3 ± 1.2	283 ± 11*	0.9 ± 0.9
**Sham + okadaic acid + mAIH**			
Baseline	43.8 ± 1.5	313 ± 10	1.3 ± 0.6
Hypoxia	44.0 ± 1.3	40 ± 2.4*	0.1 ± 0.5
30 min	43.9 ± 0.8	249 ± 16*	1.6 ± 0.67
60 min	43.9 ± 1.4	251 ± 22*	0.6 ± 0.4
90 min	43.8 ± 0.9	240 ± 26*	− 0.4 ± 0.7
**LPS + okadaic acid + mAIH**			
Baseline	40.5 ± 1.0	317 ± 7.8	0.7 ± 0.6
Hypoxia	41.2 ± 1.1	40 ± 1.3*	2.0 ± 0.3
30 min	40.6 ± 1.0	268 ± 14*	2.4 ± 0.3
60 min	40.6 ± 0.7	268 ± 11*	2.1 ± 0.3
90 min	41.2 ± 0.8	261 ± 12*	1.6 ± 0.5

Values are means ± SE. Sham refers to group of rats that were injected with saline (i.p.) 24 h before start of experiments. Similarly, in different group of rats, LPS was also given 24 h before experiments began. Vehicle (aCSF) or okadaic acid solution were delivered intrathecally at the cervical spinal cord

*PaCO*_*2*_ arterial CO_2_ pressure, *PaO*_*2*_ arterial O_2_ pressure, *SBEc* standard excess base

*Represents a significant difference compared to baseline (*P* < 0.05)

**Table 2:** Physiological variables at baseline and for 90 min post-intrathecal injections of either the vehicle or okadaic acid in time-control recordings

Experimental groups	PaCO_**2**_ (mmHg)	PaO_**2**_ (mmHg)	SBEc mmol/L
**Sham + vehicle time control**			
Baseline	42.5 ± 1.2	304 ± 9	1.4 ± 0.5
30 min	43.1 ± 1.1	278 ± 13*	2.4 ± 0.5
60 min	42.4 ± 1.2	288 ± 10*	2.5 ± 0.5
90 min	42.9 ± 0.9	289 ± 7*	2.6 ± 0.6
**LPS + vehicle time control**			
Baseline	42.7 ± 0.6	318 ± 7	1.6 ± 0.4
30 min	43.0 ± 0.5	306 ± 9	1.8 ± 0.5
60 min	42.1 ± 0.6	303 ± 10	1.3 ± 0.6
90 min	42.7 ± 0.4	301 ± 8	1.7 ± 0.5
**Sham + okadaic acid time control**			
Baseline	40.9 ± 0.8	315 ± 5	0.20 ± 0.6
30 min	41.5 ± 0.6	309 ± 6	1.4 ± 0.5
60 min	41.2 ± 0.8	304 ± 7	1.0 ± 0.6
90 min	40.0 ± 0.9	290 ± 10*	− 0.4 ± 0.9
**LPS+ okadaic acid time control**			
Baseline	45.7 ± 2.2	279 ± 11	1.6 ± 0.4
30 min	45.6 ± 2.4	270 ± 9	1.8 ± 0.5
60 min	45.6 ± 2.1	258 ± 12	1.3 ± 0.6
90 min	45.8 ± 2.3	245 ± 20	1.7 ± 0.6

Values are means ± SE. Sham refers to group of rats that were injected with saline (i.p.) 24 h before start of experiments. Similarly, in different group of rats, LPS was also given 24 h before experiments began. Time control recordings are from rats that did not get exposed to intermittent hypoxia

*PaCO*_*2*_ arterial CO_2_ pressure, *PaO*_*2*_ arterial O_2_ pressure, *SBEc* standard excess base

*Represents a significant difference compared to baseline (*P* < 0.05)

**Table 3:** Arterial pressure and respiratory frequency in sham or LPS-injected rats receiving either spinal intrathecal vehicle or okadaic acid

Experimental groups	Arterial pressure (mmHg)	Respiratory frequency
**Sham + vehicle + mAIH**		
Baseline	127 ± 6	53 ± 2
30 min	105 ± 5*	53 ± 3
60 min	96 ± 7*	54 ± 2
90 min	91 ± 5*	54 ± 2
**LPS + vehicle + mAIH**		
Baseline	131 ± 9	55 ± 3
30 min	113 ± 10*	49 ± 5
60 min	106 ± 11*	50 ± 4
90 min	102 ± 10*	49 ± 4
**Sham + okadaic acid + mAIH**		
Baseline	117 ± 4	52 ± 2
30 min	104 ± 6*	51 ± 1
60 min	97 ± 6*	54 ± 1
90 min	96 ± 7*	54 ± 1
**LPS + okadaic acid + mAIH**		
Baseline	114 ± 10	51 ± 2
30 min	102 ± 7	50 ± 2
60 min	100 ± 5	52 ± 2
90 min	98 ± 5	52 ± 2
**Sham + vehicle time control**		
Baseline	115 ± 6	50 ± 5
30 min	108 ± 10	49 ± 3
60 min	98 ± 9	49 ± 5
90 min	99 ± 11	46 ± 2
**LPS + vehicle time control**		
Baseline	117 ± 12	50 ± 3
30 min	96 ± 12	48 ± 4
60 min	91 ± 13	46 ± 4*
90 min	90 ± 11	46 ± 3*
**Sham + okadaic acid time control**		
Baseline	104 ± 6	49 ± 2
30 min	98 ± 5	50 ± 2
60 min	92 ± 6	49 ± 2
90 min	84 ± 6*	48 ± 3
**LPS + okadaic acid time control**		
Baseline	113 ± 12	48 ± 2
30 min	98 ± 12	49 ± 2
60 min	99 ± 10	48 ± 3
90 min	94 ± 11	48 ± 2

Values are means ± SE. Data represents values at baseline and at 30, 60, and 90 min post hypoxia, or at equivalent time points in time-matched control recordings (i.e., no hypoxia). Sham refers to group of rats that were injected with saline (i.p.) 24 h before start of experiments. Similarly, in different group of rats, LPS was also given 24 h before experiments began

*PaCO*_*2*_ arterial CO_2_ pressure, *PaO*_*2*_ arterial O_2_ pressure, *SBEc* standard excess base *Represents a significant difference compared to baseline (*P* < 0.05)

To quantify fluorescence intensities for molecules of interest in the region of CtB-labeled phrenic motor neurons, images were analyzed using a custom-written MATLAB algorithm (MathWoks, Natick, MA, USA). The algorithm identified CtB-labeled phrenic motor neuron soma and quantified signal intensity of phospho-ERK1/2 and phospho-p38MAPK immunoreactivity in each CtB-labeled soma and defined region of interest around phrenic motor neurons. Methods for protein-specific signal intensity quantification within CtB-labeled phrenic motor neuron soma have been previously described [55]. CtB-labeled phrenic motor neurons were located within the ventral horn of the cervical spinal cord using a custom adaptive thresholding algorithm in MATLAB (MathWorks, Natick, MA, USA). The adaptive threshold was calculated by constructing a pixel intensity histogram from the image. First, a pixel intensity histogram is constructed within a circle (diameter = 100 μm) containing the CtB-positive region of the ventral horn. The pixel intensity corresponding to 95th percentile value was used as the threshold value across all images to determine phrenic motor neurons. Selection of a fixed percentile threshold returns a higher threshold value for an image with high signal and background intensities and a lower threshold value for an image with low signal and background intensities. CtB images were binarized using the adaptive threshold; thus, CtB-positive areas were assigned the value of unity, whereas CtB-negative areas were set to zero. The center of gravity representing the center of phrenic nucleus in a given image was calculated using the binarized CtB image as previously discussed [55]. Since phospho-ERK1/2 immunolabeling is localized at the extrasomatic areas of phrenic motor nucleus, intensity of the pERK labeling was calculated by averaging the pixel intensities within the phrenic nucleus (i.e., a circular region of interest with a diameter of 100 μm, centered around the center of gravity calculated from binarized CtB images earlier). Background intensity was calculated as the median value of the circular region and subtracted from the averaged pixel intensity value.

For phospho-p38 MAPK quantification, the pixel intensity corresponding to the 95th percentile was selected as the adaptive threshold to account for changes in CtB fluorescence intensities across animals and images. Using a fixed percentile threshold value would yield a higher threshold in an image with brighter signal and background fluorescence intensities, or a lower threshold in an image with dimmer signal and background fluorescence intensities. The pixels above the adaptive threshold (95th percentile across all pixel intensities) were considered CtB-positive. The coordinates of CtB-positive pixels/areas were used to measure fluorescence intensities of phospho-p38 MAPK. Final fluorescence intensities were determined after subtraction of local background labeling by determining the median value. Protein quantification in a defined region of interest has been previously described in detail [55]. For statistics, analyses of multiple comparisons were performed by ANOVA with Tukey significant difference test as post hoc test and analyses of single comparisons were performed by *t* test.

## Results

### Systemic LPS blocks phrenic and hypoglossal long-term facilitation (LTF)

**Fig. 1:**
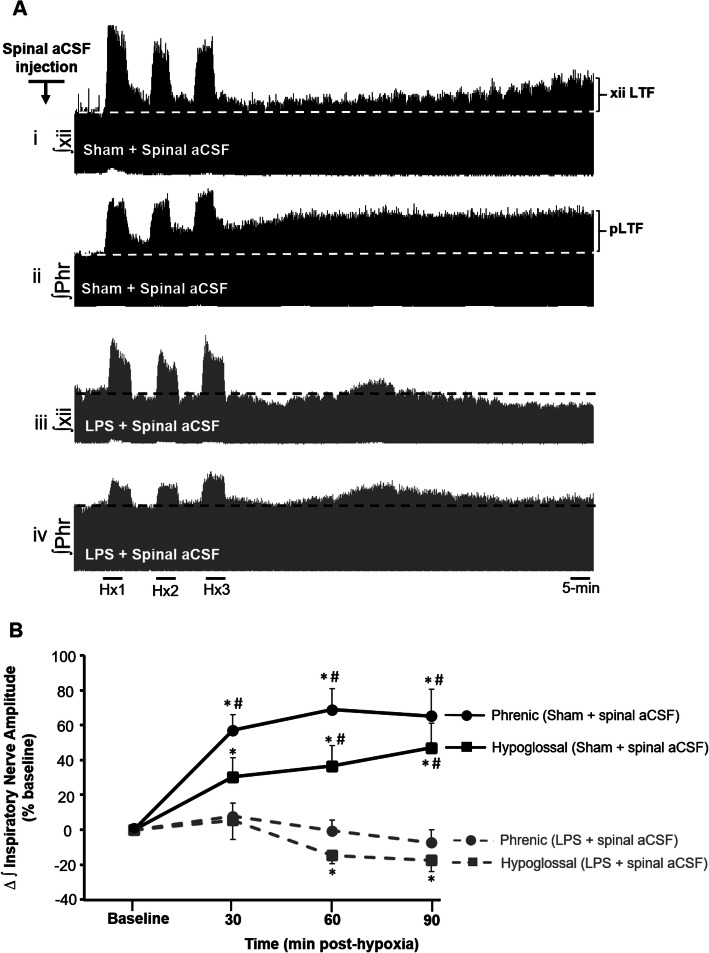
Systemic LPS prevents mAIH-induced long-term facilitation (LTF) of inspiratory hypoglossal and phrenic motor output. **a** Representative integrated inspiratory phrenic (Phr) and hypoglossal (xii) neurograms before, during and for 90 min after exposures to mAIH in sham-vehicle (i and ii) or LPS-treated rats (iii and iv). Top two traces (i and ii) are from one rat, demonstrating respiratory LTF of phrenic and hypoglossal motor output. Bottom two (iii and iv) traces are from a separate rat illustrating lack of LTF following systemic LPS treatment. **b** Mean values expressed as a percentage change from baseline (baseline = 0), showing that mAIH triggers LTF in sham-vehicle-treated rats. In rats pre-treated with systemic LPS, LTF was absent. Values are normalized means ± SE. *Significant difference compared to baseline; ^#^significant difference compared to LPS-treated group at the indicated time points: for all, *p* < 0.05. mAIH, moderate acute intermittent hypoxia; Hx1, Hx2, Hx3, hypoxic episodes 1, 2, and 3; Phr, phrenic; xii, hypoglossal; pLTF, phrenic long-term facilitation; xiiLTF, hypoglossal long-term facilitation

Typical integrated phrenic and hypoglossal nerve recordings before, during, and after mAIH exposures are shown in Fig. 1. In sham vehicle-treated rats that did not receive LPS, phrenic and hypoglossal nerve burst amplitudes progressively increased following mAIH, exhibiting significant augmentation above baseline by 90 min post-hypoxia (65 ± 15% and 46 ± 14% increase in inspiratory phrenic and hypoglossal nerve burst amplitudes at 90 min, respectively, *p* < 0.05; *n* = 6). This confirmed respiratory LTF expression in both brainstem hypoglossal and spinal phrenic motor pools. In a separate rat group pre-treated with LPS, mAIH failed to trigger LTF in either nerve; phrenic and hypoglossal nerve burst amplitudes remained near baseline levels throughout the post-hypoxic period. At 90 min post-hypoxia, the change in integrated phrenic burst amplitude was 7 ± 7.3% below baseline (*p* = 0.34; *n* = 7), a response significantly lower than rats injected with vehicle (*p* < 0.05 in the overall ANOVA; Fig. 1a, b). Similar effects were observed in hypoglossal activity which remained significantly depressed 90 min post-hypoxia in LPS-treated rats versus sham vehicle-treated rats (46 ± 14% above baseline versus 18 ± 6 % below baseline at 90-min post-mAIH in sham vehicle and LPS-treated rats, respectively, p < 0.05; Fig. 1a, b). These results confirm previous reports that systemic LPS blocks pLTF expression, and are the first demonstration that LPS also blocks LTF in supra-spinal respiratory (i.e., brainstem hypoglossal) motor pools. We also compared respiratory frequency during baseline and during the post-hypoxic period (Table 3). Baseline breathing frequency was the same in sham-vehicle and LPS-treated rats and exhibited no time-dependent differences during the post-hypoxic period (*p* = 0.331 for comparison between treatments).

### Cervical spinal okadaic acid delivery restores phrenic LTF in LPS-treated rats

**Fig. 2:**
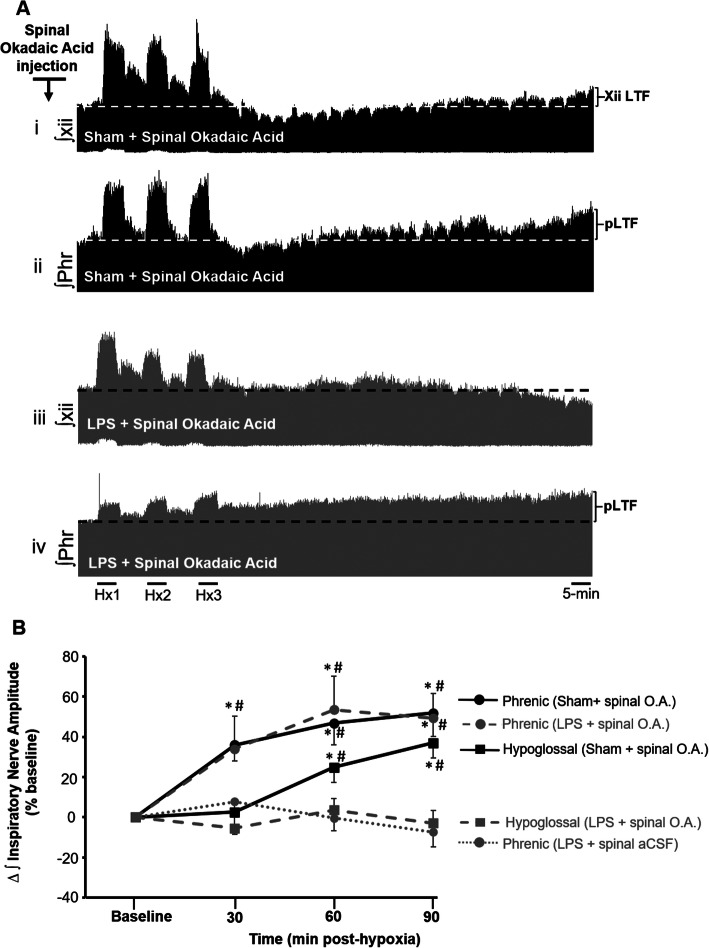
Systemic LPS prevents phrenic long-term facilitation (LTF) via activity of cervical spinal okadaic acid-sensitive serine/threonine protein phosphatases. **a** Representative integrated inspiratory phrenic (Phr) and hypoglossal (Xii) neurograms before, during and for 90 min after moderate acute intermittent hypoxia (mAIH) in a sham-vehicle (i and ii) and a LPS-treated rat (iii and iv) receiving cervical spinal okadaic acid. While cervical spinal okadaic acid administration did not interfere with the development of LTF in the sham-vehicle treated rat (i and ii) the same intervention restored phrenic LTF in rats that had received LPS (iv). **b** Mean group data demonstrating that by 90 min post hypoxia, inspiratory phrenic and hypoglossal nerve amplitude was significantly enhanced above baseline levels (baseline = 0) in sham vehicle treated rats receiving cervical spinal okadaic acid. In rats pre-treated with systemic LPS, cervical spinal okadaic acid restored phrenic LTF, whereas as expected, hypoglossal nerve amplitude remained near baseline levels throughout the 90-minute recording period. Values are normalized means ± SE. *Significant difference compared to baseline; ^#^significant difference compared to respective LPS-treated group at the indicated time point: for all, *p* < 0.05. Hx1, Hx2, Hx3, hypoxic episodes 1, 2, and 3; Phr, phrenic; xii, hypoglossal; pLTF, phrenic long-term facilitation; xiiLTF, hypoglossal long-term facilitation; O.A., okadaic acid

We tested the hypothesis that systemic LPS disrupts LTF by a mechanism that requires okadaic acid-sensitive serine-threonine protein phosphatase 1/2A activity. This was tested by delivering okadaic-acid to the intrathecal space of the cervical spinal cord. Before doing so however, we characterized the effects of intrathecal okadaic acid on mAIH-induced plasticity without inflammation. This was important for making comparisons with LPS pre-treated rats receiving spinal okadaic acid. Our results demonstrate that cervical spinal okadaic acid had no detectable impact on LTF development in sham vehicle-treated rats: at 90 min following mAIH, phrenic and hypoglossal nerve amplitudes had significantly increased to 51 ± 11% and 37 ± 7% above baseline levels, respectively (*p* < 0.05 for both; Fig. 2a, b; *n* = 6 ). The magnitudes of hypoglossal and phrenic LTF were not statistically different from sham rats receiving cervical spinal injections of vehicle, demonstrating that okadaic acid has minimal impact on the normal expression of mAIH-induced LTF (*p* > 0.05 in the overall ANOVA post hoc test for both phrenic and hypoglossal). There was no time-dependent change in respiratory frequency following mAIH (*p* > 0.05 at every time points post-hypoxia relative to baseline; Table 3)

Once we characterized the effect of spinal okadaic acid on LTF expression in sham vehicle control rats, we then asked if the same intervention restores pLTF in rats pre-treated with systemic LPS. Indeed intrathecal okadaic acid restored pLTF: at 90 min post-mAIH, inspiratory phrenic nerve burst amplitude was significantly increased by 49 ± 12% above baseline, confirming pLTF restoration in LPS-treated (*p* = 0.002; Fig. 2a (iv), b; *n* = 6). At 90 min post-mAIH, pLTF magnitude was not significantly different from any sham vehicle-treated rat group (no difference compared to sham vehicle + mAIH plus or sham vehicle + okadaic acid + mAIH) confirming that pLTF was fully restored by spinal serine/threonine PP inhibition despite the fact that animals had received systemic LPS (*p* > 0.05 in the overall ANOVA).

As expected, hypoglossal LTF was still absent in LPS-treated rats that received spinal okadaic acid since this pharmacological manipulation was performed caudally, and drug distribution to the brainstem was not expected as shown previously (Baker-Herman and Mitchell, 2002). This observation further confirms that LPS-induced mechanisms are operating at brainstem containing hypoglossal motor neurons (3 ± 6% below baseline at min-90 post hypoxia, *p* > 0.05; Fig. 2 A (iii) and 2B). Thus, we conclude that systemic LPS prevents mAIH-induced spinal, as well as brainstem respiratory motor plasticity, and it does so at least in part via recruitment of okadaic acid-sensitive serine-threonine PP activity.

### Respiratory activity is stable in time control rats

Inspiratory phrenic and hypoglossal nerve activities were quantified in separate rat groups without mAIH. These rats received systemic vehicle or LPS, combined with either spinal intrathecal vehicle or okadaic acid administration (sham vehicle + spinal vehicle, *n* = 5; sham vehicle + spinal O.A., *n* = 6; LPS + spinal vehicle, *n* = 6; LPS + spinal O.A, *n* = 6). There was no time-dependent change in phrenic or hypoglossal burst amplitude at any time point during the recording period in any group (*p* > 0.05 in the over-all ANOVA; data not shown). Thus, stability of phrenic nerve activities in our time control experiments further ensured that mAIH-induced LTF of phrenic amplitude was indeed due to plasticity triggered by hypoxia rather than spontaneous enhancement in inspiratory nerve amplitude over time per se (i.e., “drift”).

### Baseline physiological measurements

**Fig. 3:**
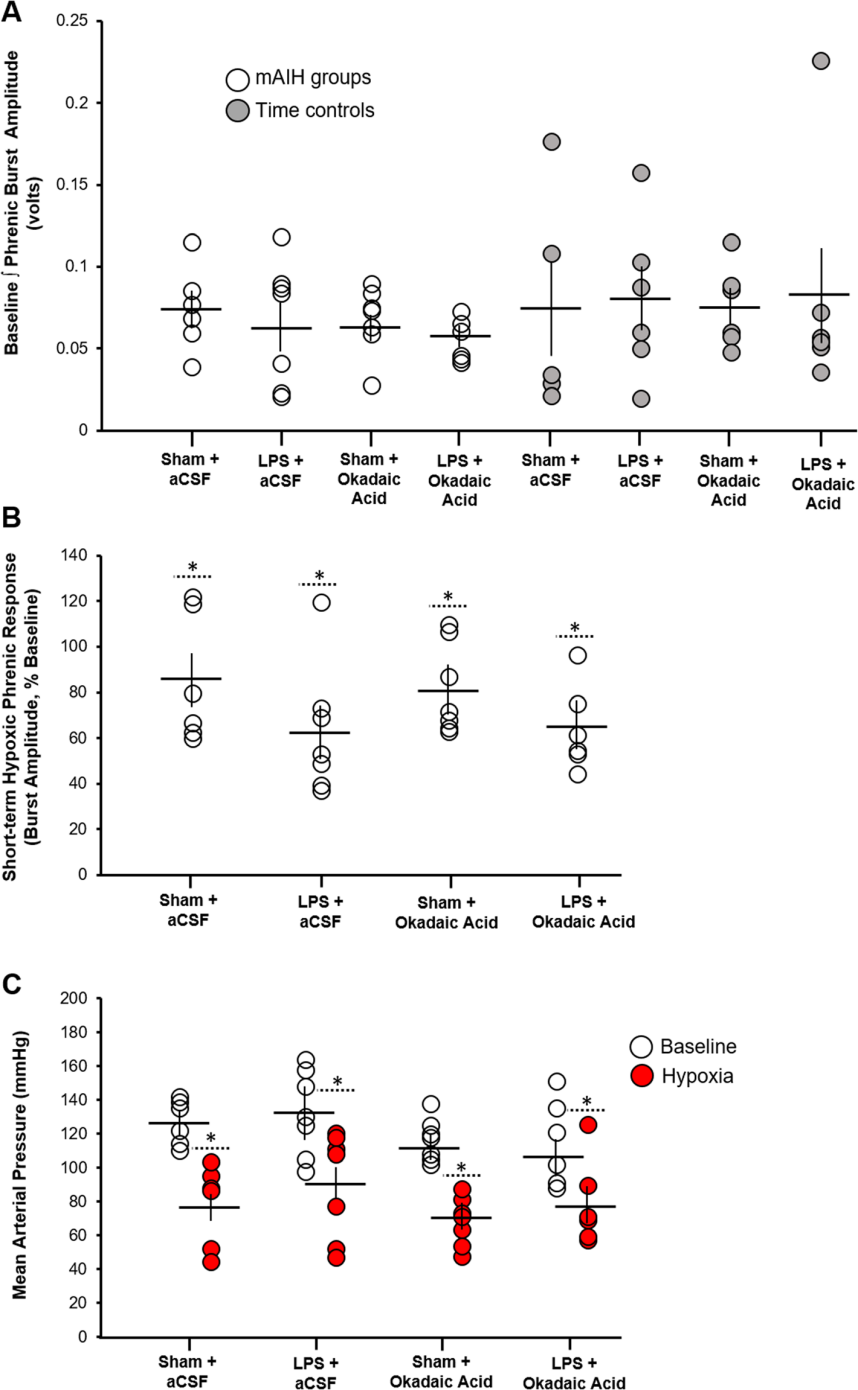
Baseline inspiratory phrenic burst amplitude, short-term hypoxic phrenic response, and mean arterial pressure in different treatment groups. **a** Group data demonstrating that baseline phrenic nerve inspiratory amplitude is similar in all treatment groups. **b** Normalized, inspiratory phrenic nerve burst amplitudes were similarly enhanced within hypoxic episodes in groups exposed to moderate acute intermittent hypoxia (mAIH). There was no significant difference among the groups. **c** Mean arterial blood pressure (mmHg) during baseline conditions and hypoxic episodes (red). Hypoxia significantly decreased mean arterial pressure in all groups to the same degree. Solid horizontal line within each data series indicates average group mean, and the vertical bar protruding above and below the horizontal line indicates standard error for each data set. Circles indicate individual data points in each experimental group. *Significant difference versus baseline values with *p* value < 0.05

Baseline phrenic nerve burst amplitude was compared among the various experimental groups and we determined that there was no significant difference in the overall comparison (*p* = 0.653 in the overall ANOVA, Fig. 3a). This observation demonstrates that normalization as a percentage change from baseline was appropriate to compare changes in the magnitude of phrenic nerve activity (see sections above). Further, it demonstrates that none of the vehicle or LPS injections affected basal phrenic motor output as measured during neurophysiological recordings. Therefore, the differences in the magnitude of pLTF between experimental groups were unlikely to arise from baseline amplitude differences causing a “ceiling” effect, muting the potential plastic response to intermittent hypoxia.

We also compared the short-term hypoxic phrenic response in all groups exposed to moderate AIH (Fig. 3b). As expected, hypoxia triggered a robust and significant increase in phrenic burst amplitude in all groups tested (*p* < 0.05 comparing hypoxic phrenic response versus baseline in each group). However, we found no significant group difference in the phrenic hypoxic response since phrenic burst amplitude was increased to the same degree in all groups (*p* = 0.267 in the overall ANOVA). We next compared mean arterial pressure (MAP) during baseline, as well as during hypoxic episodes (Fig. 3c). No difference in baseline MAP was detected (*p* = 0.337 in the overall ANOVA). As expected, there was a significant decrease in MAP during hypoxic episodes within each group, but this drop in MAP was similar comparing the groups tested (*p* = 0.414 in the overall ANOVA comparing BP during hypoxia across all groups). Together, these observations demonstrate that systemic LPS at dose employed here did not have any discernable effect on baseline phrenic motor activity, short-term hypoxic phrenic responses, or blood pressure responses during hypoxia.

### LPS prevents mAIH-induced ERK1/2 MAPK phosphorylation

Serine/threonine protein phosphatases including PP1 and PP2A inhibit ERK1/2 MAPK activity by dephosphorylation of specific threonine residues of the ERK enzyme [45–47, 56]. In the present study, cervical spinal okadaic acid-sensitive protein phosphatases were shown to impose an important constrain on ERK-dependent pLTF following systemic LPS (Fig. 2a, b). However, it is unknown whether such restrain involves deficits in ERK1/2 MAPK phosphorylation/activation. Using quantitative optical density immunofluorescence quantification, we determined if LPS affects mAIH-induced phosphorylation of ERK 1/2 MAPK.

**Fig. 4:**
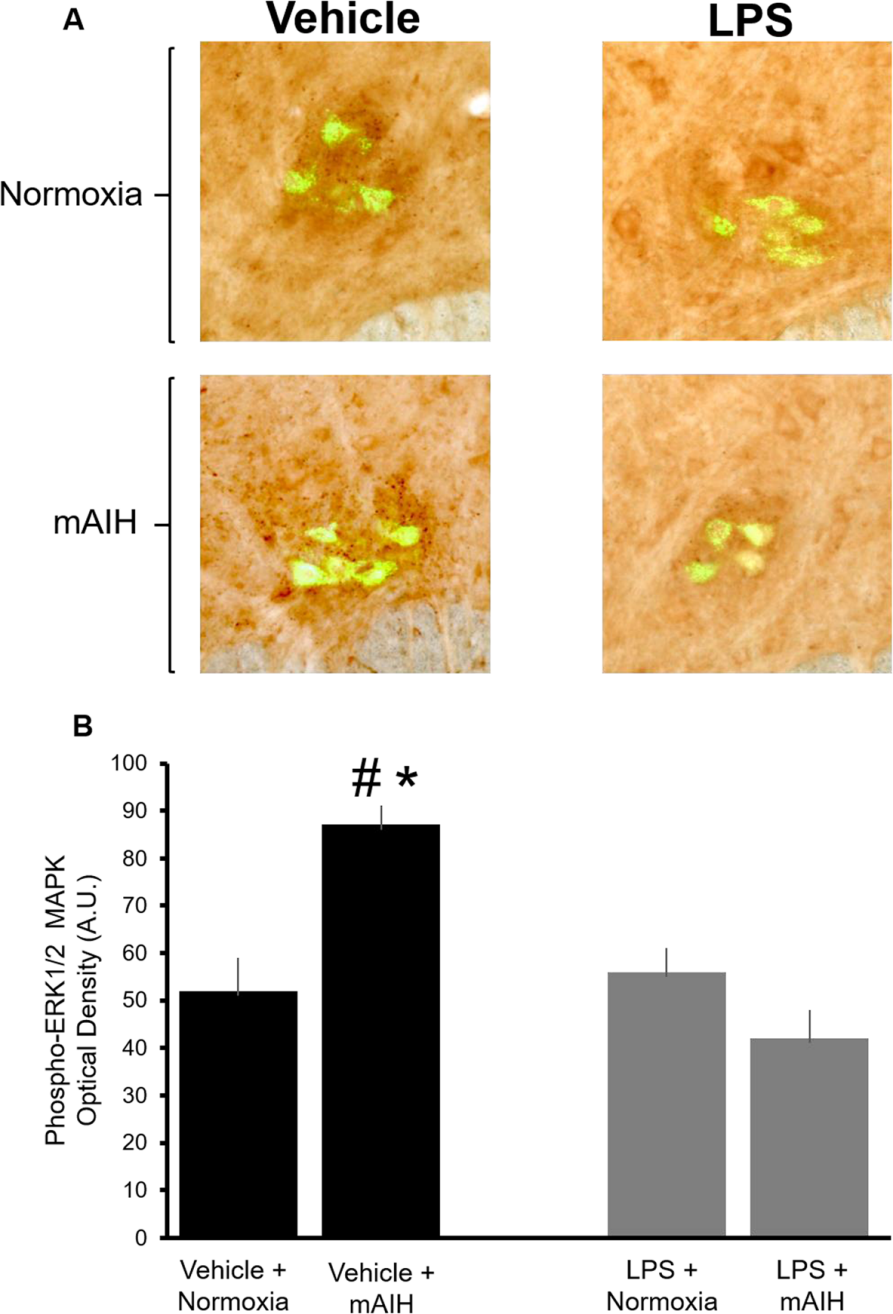
Systemic LPS blocks hypoxia-induced phosphorylation of ERK1/2 MAPK. **a** Double immunofluorescence staining of phosphorylated ERK1/2 MAPK (dark brown puncta) with the retrograde tracer (green) Cholera toxin B fragment (CtB) in phrenic motor neurons in the ventral horn of the cervical spinal cord. Representative × 20 magnification of the C4 segment of the spinal cord reveals minimal staining for phospho ERK1/2 MAPK on/near phrenic motor neurons in a vehicle treated rat that was not exposed to hypoxia. In contrast, phospho ERK1/2 MAPK staining appeared more intense in response to moderate acute intermittent hypoxia (mAIH) relative to normoxic controls. When pre-treated with LPS however, mAIH no longer appeared to increase ERK1/2 MAPK phosphorylation levels. **b** Average group data demonstrating that mAIH causes a significant enhancement in phosphorylated ERK1/2 MAPK levels on/near phrenic motor neurons. In contrast, mAIH failed to enhance phosphorylated ERK1/2 MAPK levels in rats pre-treated with LPS. Data are means ± S.E. *Significant difference from vehicle treated normoxia group and # indicates significant difference compared to all other groups: for all, *p* < 0.05. A.U., arbitrary units

In vehicle-treated rats not exposed to mAIH, phospho-ERK1/2 immunofluorescence staining suggested a low basal constitutive expression in cholera toxin B-labeled phrenic motor neurons (Fig. 4a, b; *n* = 6). In contrast, rapid and significant increases in phospho-ERK1/2 levels occurred in phrenic motor neurons following mAIH (67 ± 3.5% increase above baseline levels; *p* = 0.0056; *n* = 5). This finding demonstrates that mAIH normally triggers a rapid increase in phosphorylated ERK1/2 levels within phrenic motor neurons, consistent with its necessary role in mAIH-induced pLTF. After LPS delivery, phospho-ERK1/2 immunofluorescence still showed low constitutive expression within phrenic motor neurons: basal expression (without mAIH exposure; *n* = 6) of phospho-ERK1/2 was the same comparing vehicle and LPS-treated rats (52 ± 7 versus 56 ± 6 basal optical intensity strength in vehicle and LPS-treated rats, respectively; *p* > 0.05). However, the impact of LPS on ERK1/2 MAPK activation became clear when examining the effect of mAIH in LPS-treated rats. In contrast to sham control rats, mAIH no longer increased ERK1/2 phosphorylation following LPS treatment (*n* = 6). In fact, ERK1/2 phosphorylation remained close to baseline values following mAIH versus LPS-treated rats that did not receive mAIH (56 ± 6 versus 43 ± 5 optical density strength in LPS without hypoxia versus LPS with mAIH respectively; *p* = 0.152). Taken together, our findings are consistent with the idea that LPS inhibits phrenic motor plasticity by preventing mAIH-induced ERK 1/2 MAPK activation within phrenic motor neurons.

### Systemic LPS enhances p38 MAPK phosphorylation in phrenic motor neurons

**Fig. 5:**
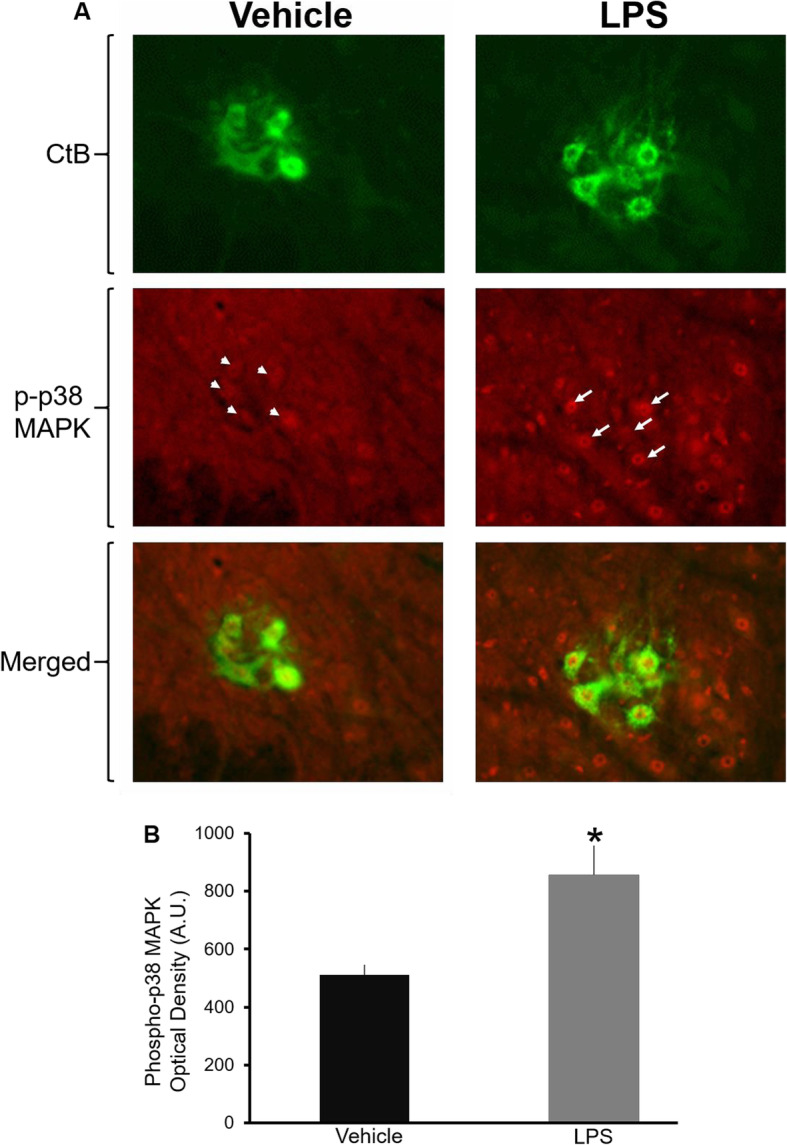
Systemic LPS enhances p38-MAPK phosphorylation in phrenic motor neurons. **a** Double immunofluorescent staining illustrating phosphorylated p38-MAPK (red) with the retrograde tracer (green) Cholera toxin B fragment (CtB) in phrenic motor neurons in the ventral horn of the cervical spinal cord. Representative × 20 magnification of the C4 segment of the spinal cord reveals minimal staining for phospho p38-MAPK (arrow heads) in phrenic motor neurons in a vehicle treated rat. In contrast, phospho p38-MAPK staining is enhanced in phrenic motor neurons (and non-phrenic cells) following LPS treatment (arrows). **b** Average group data demonstrating that systemic LPS significantly enhances phosphorylated p38-MAPK levels within CtB-positive phrenic motor neurons as measured by optical density analysis. Data are means ± S.E. *p < 0.05 indicating significant difference compared to vehicle treated rats. A.U., arbitrary units

We showed that LPS blocks pLTF by a mechanism that requires spinal okadaic acid-sensitive PP activity and mAIH-induced increase in ERK1/2 MAPK phosphorylation is inhibited by LPS (see above). Our next aim was to connect the dots, seeking correlative evidence that deficits in the ERK-dependent pLTF pathway may be paralleled by activation of p38 MAPK—a well-characterized MAPK-mediated pathway traditionally known to oppose the actions of ERK MAPK. To determine whether LPS changes p38 MAPK phosphorylation levels, we evaluated LPS effects on dually phosphorylated (enzymatically activated) p38 MAPK expression in phrenic motor neurons using optical density immunofluorescence (Fig. 5a, b). Results were compared to sham vehicle control rats. In sham vehicle control rats, weak phospho-p38 MAPK staining was visible in the ventral horn of the cervical spinal cord: although minimal, some staining was colocalized within cholera toxin B-labeled phrenic motor neurons. After LPS however, more phrenic motor neurons were positive for phospho-p38 MAPK, and the staining intensity within these cells had significantly increased (~ 40% increase in p38MAPK staining in LPS-treated rats versus vehicle sham controls, Fig. 5b; *p* = 0.032; *n* = 6). Increased phospho-p38 MAPK was evident in cells other than phrenic motor neurons, suggesting that LPS-induced systemic inflammation causes a wide-spread increase in p38 MAPK signaling in the cervical spinal ventral horn. These cells were not specifically identified.

## Discussion

In this study, we confirm that 24 h following systemic LPS (100 μg/kg, i.p.), mAIH-induced pLTF is blocked. We also show that systemic LPS inhibited LTF of upper airway hypoglossal motor output, demonstrating that detrimental effects of LPS on the expression of respiratory motor plasticity are widespread. LPS increased phosphorylated p38 MAPK levels within phrenic motor neurons, indicative of enhanced p38 MAPK activity. In support of our hypothesis, cervical spinal intrathecal pre-treatment with okadaic acid, a serine/threonine PP 1/2A inhibitor, restored pLTF in LPS-treated rats. Thus, okadaic acid-sensitive serine/threonine protein phosphatases play an essential role in constraining pLTF expression with systemic inflammation. Lastly, mAIH failed to enhance phrenic motor neuron ERK1/2 MAPK phosphorylation with LPS treatment, linking ERK 1/2 MAPK phosphorylation with molecules proposed to negatively regulate phrenic motor plasticity during systemic inflammation.

### Systemic LPS abolishes phrenic and hypoglossal long-term facilitation

Although LPS does not readily cross the blood-brain barrier, numerous lines of evidence demonstrate that it can induce neuroinflammation in the central nervous system [57], including the spinal cord [23]. In this study, we confirm that even a low-dose, systemic LPS blocks the expression of mAIH-induced spinal (phrenic) respiratory motor plasticity and extend our knowledge by demonstrating that mAIH-induced brainstem (hypoglossal) respiratory motor plasticity is also impaired. To our knowledge, this is a first demonstration that a systemic inflammatory insult can occlude the expression of motor plasticity within brainstem respiratory circuits in vivo. Hypoglossal LTF represents one potential mechanism to increase upper airway tone, thereby preserving upper airway patency and reducing collapse of the upper airways [58–60]. If true, loss of hypoglossal LTF in people with ongoing systemic/neuroinflammation may further predispose individuals to repeated airway collapse. A better understanding of cellular mechanisms regulating hypoglossal motor plasticity may guide development of novel therapeutic strategies for the preservation of airway tone. Factors such as neuroinflammation that mitigate plasticity in spinal and brainstem motor circuits must be carefully considered when designing therapeutic strategies to maintain/restore breathing capacity or stability in individuals with breathing impairments [61–63].

### Moderate acute intermittent hypoxia enhances phrenic ERK1/2 MAPK phosphorylation

ERK 1/2 MAPK integrates inputs from several transmembrane proteins, including serotonergic, cholinergic, adrenergic, dopaminergic, glutamatergic, and neurotrophin receptors. In doing so, it can influence wide range of cellular functions such as gene expression, protein synthesis, dendritic spine stabilization, modulation of ion channels, and receptor insertion [64]. By activating gene expression machinery, ERK signaling regulates the expression of several plasticity-related proteins including BDNF [65–68]. Thus, it can play a crucial role in several forms of synaptic plasticity [69–71]. As demonstrated here, the rapid enhancement of ERK1/2MAPK phosphorylation in phrenic motor neurons following mAIH is consistent with a major role in the induction of neural plasticity. This parallels our previous neurophysiological demonstrations that cervical spinal inhibition of ERK1/2 MAPK signaling blocks serotonin-dependent, mAIH-induced pLTF [31]. Although in the present study we did not investigate the subcellular localization of phosphorylated ERK1/2 MAPK in detail, phospho-ERK appeared localized in regions immediately surrounding phrenic motor neuron somata, possibly in dendrites or presynaptic terminals. It was minimally expressed within cytoplasm (or nucleus) of CtB-positive phrenic motor neuron somata. Although we could not determine pre- versus post-synaptic localization with the methods employed, the localization of puncta surrounding the phrenic motor neurons is consistent with the idea that ERK1/2 MAPK plays a role in modulation of synaptic inputs onto phrenic motor neurons or processes located in the distal dendrites such as persistent inward currents, known to decrease the dendritic space constant [72]. Since ERK is necessary for phrenic motor facilitation elicited by serotonin type 2A receptors [29], and serotonin 2A receptors are expressed in the dendrites of phrenic motor neurons (Allen & Mitchell, unpublished observations), phosphorylated ERK in the distal dendrites is consistent with a role in serotonin 2A contributions to phrenic motor facilitation following mAIH. Additional studies are needed to test these hypotheses.

### LPS effects on hypoxia-induced phosphorylation of ERK1/2 MAPK: impact on plasticity

One major finding of this study is the demonstration that systemic LPS hindered ERK1/2 MAPK phosphorylation in response to mAIH. This finding was confirmed using immunofluorescent optical density analysis, whereby mAIH failed to increase ERK1/2 MAPK phosphorylation in the phrenic motor nucleus of LPS-treated rats. This was a fundamental observation since it demonstrated the impact of systemic inflammation on the phosphorylation of a key enzyme necessary for phrenic motor plasticity. Since our immunofluorescent optical density analysis demonstrated that LPS prevented increased ERK1/2 MAPK phosphorylation in response to mAIH, we suggest that LPS activated signaling mechanisms which undermined this key step necessary for enzyme activation.

Both phospho-tyrosine and phospho-threonine are involved in ERK1/2 MAPK activation, and both can be dephosphorylated by PP2A and/or PP1—protein phosphatases specific for serine and threonine residues [73, 74]. ERK1/2 MAPK is functionally active only when both tyrosyl and threonyl residues are phosphorylated [56, 75]. In this study, we used an antibody that detects ERK1/2 when phosphorylated at both tyrosyl and threonyl residues, or singly phosphorylated at a threonyl residue. Thus, basal phosphorylation might reflect single or dually phosphorylated enzyme. With this limitation in antibody detection (single versus dual), we cannot conclude with certainty which residues were phosphorylated by mAIH, nor can we differentiate between ERK1 versus ERK2. However, the observation that systemic LPS inhibited pLTF via activity of serine-threonine phosphatases is consistent with the idea that pLTF inhibition results from de-phosphorylation of ERK1/2 MAPK at threonine residues.

Serine/threonine protein phosphatases may act on the ERK signaling at multiple levels. For example, PP1 and/or PP2A may act on upstream activators of ERK1/2 MAPK. Some studies have demonstrated that serine-threonine protein phosphatases can inactivate MEK—the immediate upstream activator of ERK [46]. Other studies have provided evidence that these phosphatases inhibit the ERK signaling cascade at the levels of ERK1/2 MAPK protein itself [38, 48]. Identification of specific cellular sites of action of serine/threonine protein phosphatases in the context of pLTF was beyond the scope of the present study. In addition, in this study, we did not determine whether okadaic acid restores ERK 1/2 MAPK phosphorylation following mAIH in LPS treated animals (Fig. 4). This would confirm that pLTF inhibition following LPS is a result of PP1/PP2A acting on MEK-ERK signaling (or upstream). If this were not the case, it would indicate that PPs maybe acting on other potential target molecules. This topic warrants additional studies, both to better understand mechanisms and develop effective pharmacological therapies to restore respiratory motor plasticity in the face of neuro-inflammation.

### LPS effects on p38 MAPK phosphorylation

The p38 class of MAP kinases is primarily activated through stressful extracellular stimuli and circulating cytokines and is well studied in the field of inflammation [44]. The p38 MAPK pathway is a key regulator of pro-inflammatory cytokine biosynthesis, triggering a self-sustaining cycle [76]. p38 MAPK signaling is also an important regulator of neural synaptic plasticity [67, 77, 78]. We now provide correlative evidence that systemic LPS upregulates p38 MAPK phosphorylation/activation in phrenic motor nucleus potentially orchestrating the relevant inflammatory cascades that impair mAIH-induced pLTF. This possibility aligns with a prior report from our laboratory that spinal p38 activity undermines mAIH-induced pLTF in the context of neuroinflammation induced by chronic intermittent hypoxia [49].

**Fig. 6:**
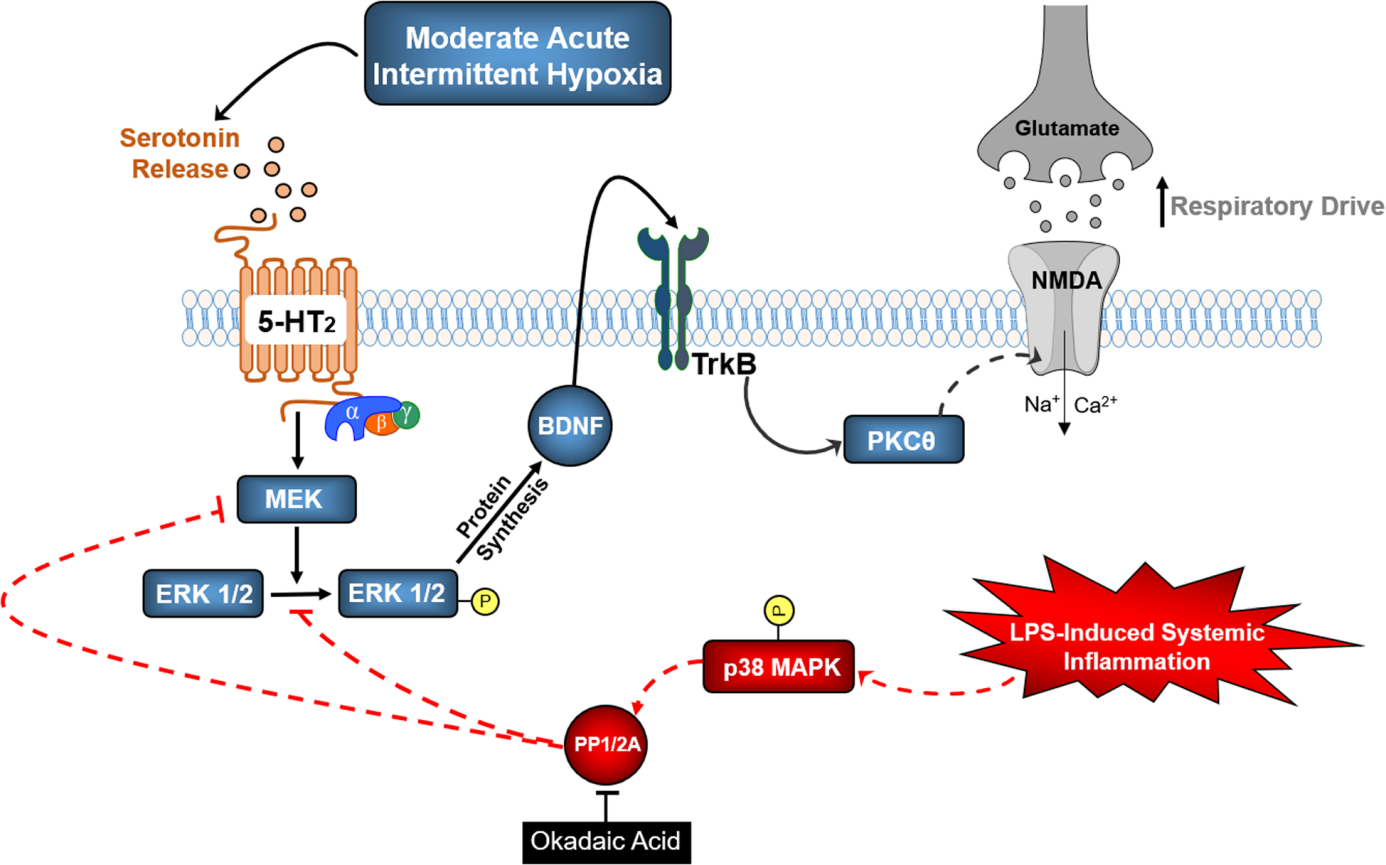
Proposed mechanism of LPS-induced inhibition of moderate acute intermittent hypoxia-induced phrenic long-term facilitation (pLTF). Moderate acute intermittent hypoxia (mAIH) induces serotonin (5-HT) release from serotonergic projections near spinal phrenic motor neurons. Serotonin activates phrenic 5-HT type 2 receptors, initiating a signaling cascade that phosphorylates ERK 1/2 MAPK and induces downstream BDNF protein synthesis. Newly synthesized BDNF signals through its high affinity receptor, TrKB, leading to increased excitatory respiratory drive onto phrenic motor neurons. Physiologically, enhanced excitatory drive is manifested as a long-lasting enhancement of phrenic motor output (i.e., pLTF). In our proposed model, systemic LPS leads to activation of inflammatory mechanisms within the central nervous system, phosphorylating (activating) p38 MAPK within phrenic motor neurons. Phospho-p38 MAPK activates okadaic acid-sensitive serine/threonine protein phosphatases (PP1/2A) that may act on 5-HT2 signaling at multiple sites. We propose that PP1/2A negatively regulates 5-HT2 signaling at the MEK and/or ERK 1/2 activation loop. Inhibition of PP1/2A activity with okadaic acid in LPS-treated rats permits 5-HT2-mediated MEK-ERK1/2 signaling, restoring mAIH-induced pLTF. Broken lines: undefined inhibitory feedback site at or below MEK level. Broken arrows: hypothesized pathway with unknown precise mechanism in the proposed model. Phosphorylation of the target molecule is marked by the letter P

Additional research is needed to understand specific p38 MAPK actions on plasticity of respiratory motor behavior following LPS-induced inflammation. However, multiple lines of evidence from literature suggest that upregulation in p38 MAPK has the potential to undermine respiratory motor plasticity following LPS. For example, there is well-documented crosstalk interaction between these opposing MAPK pathways (p38 and ERK 1/2 MAPK) in regulating synaptic strength [79]. For example, ERK 1/2 activation is required for 5-HT-induced long-term synaptic facilitation at sensorimotor synapses of Aplysia [80–82], whereas p38 MAPK activation is required for long-term depression [83, 84]. In some systems, it has been postulated that these competing pathways reciprocally inhibit one another, and the dynamic balance between them determines the direction of synaptic plasticity [85]. Crosstalk interactions between these described MAPK pathways appear to be regulated by protein phosphatase activity since p38 MAPK inhibits ERK1/2 activity via PP1 and/or PP2A [79]. As illustrated in our proposed model (Fig. 6), we hypothesize that enhanced p38 MAPK signaling negatively regulates ERK 1/2 MAPK pathway in/near phrenic motor neurons with LPS, acting indirectly via activation of okadaic acid-sensitive serine/threonine protein phosphatases. The specific identity of that protein phosphatase (i.e., PP1 versus PP2A) remains to be determined.

In this study, we explored LPS effects on two major MAPK signaling pathways, namely ERK 1/2 and p38 MAPK. Potential LPS effects on the third MAPK, c-Jun NH2-terminal kinase (JNK), have not been explored in our model system and are worth consideration. Like p38 MAPK, the JNK pathway is stimulated mainly by environmental stress and inflammatory cytokines and has profound influence on cell proliferation, apoptosis, synaptic plasticity, and learning and memory [86–90]. Several studies have demonstrated that JNK signaling negatively regulates ERK signaling [91–94]. Since ERK 1/2 activity is necessary for pLTF [31], and JNK activity negatively regulates ERK signaling in some systems, it is possible that JNK-mediated signaling participates in pLTF deficits triggered by LPS-induced inflammation. This possibility remains to be explored in future studies.

### LPS-induced impairment of respiratory motor plasticity: potential role of spinal glia

In this study, we propose that LPS-induced neuroinflammation effects physiology and signaling of phrenic motor neurons, impairing plasticity of their synaptic inputs. We demonstrate that systemic LPS prevents hypoxia-induced ERK MAPK activation within phrenic motor neurons—a key enzyme necessary for mAIH-induced pLTF [31]. We also show p38 MAPK upregulation within phrenic neurons—an enzyme known to propagate ongoing inflammation [76, 95]. While this finding suggests that the relevant cascades are occurring within phrenic motor neurons themselves, we cannot rule out additional contribution from spinal glial cells in the mechanisms by which LPS impairs pLTF.

It is well established that peripheral immune system activation induces a central nervous system (CNS) response. Important to this immune-to-CNS communication is that microglia and astrocytes propagate the inflammatory message in the brain, influencing neural network physiology [96, 97]. In this regard, microglia and astrocytes can contribute to acute phase, inflammatory and regulatory responses after a peripheral immune challenge. By inducing a variety of neuroactive substances (e.g., L-1β, IL-6, and TNFα) following an immune challenge, these glial cells can have powerful effects on cell physiology, and in particular, synaptic plasticity [98]. For example, glial cell activation contributes to maladaptive plasticity that underlies hyperalgesia following spinal injury or nerve ligation [99–104]. In contrast, inflammation-induced glial signaling undermines other forms of CNS plasticity, such as hippocampal long-term potentiation, and spinal instrumental learning [14, 105–107]. Characterization of a potential contribution by glial cells to the impairment of pLTF was beyond the scope of this study. However, given the profound effect of glial-mediated signaling on CNS neural plasticity, it remains a possibility that peripheral immune challenge by LPS may impair pLTF via mechanisms that involve spinal microglial and/or astrocytic pro-inflammatory processes. Our previous observation that systemic LPS transiently enhances cervical spinal microglial inflammatory gene expression is consistent with the idea that glia may be contributors to pLTF impairment [23]. Nevertheless, it remains to be determined whether the relevant inflammatory signaling cascades are within phrenic motor neurons, adjacent glia, or both.

## Conclusions

Our findings suggest key roles for serine/threonine protein phosphatases (PP1 and/or PP2A), ERK 1/2, and p38 MAPK in regulating hypoxia-induced phrenic motor plasticity following LPS-induced systemic inflammation. More accurate characterization of molecular mechanisms underlying inflammation effect on respiratory motor plasticity is necessary and may help develop of novel treatments and/or combinatorial therapeutic approaches to maximize respiratory motor plasticity. Such developments are highly relevant in our efforts to harness therapeutic AIH as a tool to restore breathing ability in clinical disorders that impair breathing. Understanding cellular mechanisms by which inflammation undermines respiratory motor plasticity may guide development of pharmacological therapies to maximize the functional benefit of treatments intended to harness motor plasticity as a therapeutic modality.

## Data Availability

The datasets used and/or analyzed during the current study are available from the corresponding author on reasonable request.

## Abbreviations

A.U: Arbitrary unit
aCSF: Artificial cerebral spinal fluid
CtB: Cholera toxin B
ERK1/2 MAPK: Extracellular signal-regulated kinase 1/2 mitogen activated protein kinase
i.p.: Intraperitoneal
i.v.: Intravenous
LPS: Lipopolysaccharide
mAIH: Moderate acute intermittent hypoxia
O.A: Okadaic acid
PP: Protein phosphatase
PPs: Protein phosphatases
Phr: Phrenic
pLTF: Phrenic long-term facilitation
Xii: Hypoglossal
Xii LTF: Hypoglossal long-term facilitation
